# Gut microbial metabolites as mediators of renal disease: do short-chain fatty acids offer some hope?

**DOI:** 10.4155/fsoa-2019-0013

**Published:** 2019-05-03

**Authors:** Liam M Heaney, Owen G Davies, Nicholas M Selby

**Affiliations:** 1 School of Sport, Exercise & Health Sciences, Loughborough University, Loughborough, LE11 3TU, UK; 2 Centre for Kidney Research & Innovation, School of Medicine, University of Nottingham, Derby, DE22 3DT, UK; 3 Department of Renal Medicine, Derby Teaching Hospitals NHS Foundation Trust, Derby, DE22 3NE, UK

**Keywords:** biomarkers, gut microbiome, metabolites, renal disease, short-chain fatty acids

One in ten people worldwide have chronic kidney disease (CKD), which can progress to end-stage renal disease (ESRD) and increase cardiovascular risk [1]. CKD is defined in the “Kidney Disease: Improving Global Outcomes” guidelines as abnormalities of kidney structure or function present for >3 months that have implications for health; the criteria include a reduced glomerular filtration rate, presence of albuminuria or abnormalities of kidney structure [2]. CKD is a major health concern and consumes considerable resource, with CKD-associated costs in England estimated at £1.45 billion per annum [3]. CKD has a number of different causes but there are common underlying disease mechanisms, in particular fibrosis, inflammation and hypoxia [4]. Current therapies are nonspecific and do not directly target these mechanisms. Their ineffectiveness is evident by the many patients who progress relentlessly to renal failure, whereas the high number of failed clinical trials stresses the need for a paradigm shift. Therefore, an improved knowledge and assessment of etiological factors would facilitate personalized medicine approaches by determining individual patient risk, as well as targeting of and assessing response to therapies.

One potential target is the metabolic relationship between the gut microbiome and host. Increasing evidence suggests that the gut microbiome plays an important role in homeostasis, health and disease [5]. Bacteria, as living organisms, require nutrients and energy sources to function. This demand is met via internal metabolism and often leads to the release of metabolic by-products. Importantly, these molecules cross the intestinal wall and thus enter the human circulatory system. Host diet plays a major role in bacterial metabolism, with the breakdown of dietary molecules to potentially harmful metabolites shown to associate with the development or progression of multiple diseases [5]. Renal disease patients are at a high risk of a build-up of these metabolites due to declining capabilities for systemic metabolite clearance. This build-up can lead to negative effects on host health and demonstrates an indirect effect of the gut microbiome on disease status. For instance, gut bacterial fermentation of amino acids tyrosine and tryptophan increases circulating levels of uremic toxins such as *p*-cresol (and its sulfated form) and indoxyl sulfate [6], which have been shown to be associated with progressive CKD [7,8]. Importantly, elevated levels of these metabolites have been associated with disease progression and adverse outcomes in CKD and ESRD patients [7,9,10]. In addition, trimethylamine N-oxide (TMAO) is a downstream marker of gut microbial metabolism of dietary trimethylamine-containing molecules (such as betaine, choline and L-carnitine) that has demonstrated negative mechanistic effects in models of atherosclerosis and pressure overload-induced heart failure [11,12]. Furthermore, elevated TMAO levels measured in patients with cardiovascular disease have shown strong associations with increased risk of adverse outcomes including death and rehospitalization [13,14]. Circulating TMAO levels have also been investigated in CKD, albeit to a lesser extent to date. Increased levels of TMAO have been shown to accelerate renal fibrosis in a C57BL/6J mouse model that is relatively resistant to kidney injury [15]. There are some suggestions that TMAO may also have functions as a biomarker of kidney disease, with 30-fold elevations documented in ESRD patients and elevated risk of adverse events in patients at CKD stages 3–5 that have high levels of TMAO [15–17].

However, not all metabolites produced as by-products of bacterial metabolism are associated with negative pathophysiological processes and poor clinical outcomes. In contrast to the evidence shown for uremic toxins and TMAO, short-chain fatty acids (SCFAs) are a bacterial-mediated classification of metabolites that have been associated with protective effects. SCFAs are aliphatic carboxylic acids of low carbon number (C2–6) that are produced following fermentation of dietary fiber or via protein catabolism (creating branch-chained forms), with acetate (C2), propionate (C3) and butyrate (C4) the predominant contributors to total SCFA content [18]. SCFAs have been shown to bind to G protein-coupled receptors to beneficially modulate immunity, blood pressure, inflammation, as well as fibrotic and epigenetic factors in models of kidney disease [18]. Animal and cell line supplementation studies have shown that SCFAs can ameliorate some of the progressive factors thought to underpin CKD progression through the blunting of fibrotic and inflammatory responses. Specifically, butyrate is known to reduce the production of the pro-fibrotic cytokine TGF-β1 in renal epithelial cells [19,20], as well as decreasing the expression of both pro-inflammatory (e.g., NF-κB) and pro-oxidative stress pathways (e.g. eNOS) [20,21]. Protective effects of SCFAs on fibrotic factors have included the suppression of TNF-α stimulated MCP-1 expression (observed for acetate, propionate and butyrate [22]), and the acetate-induced inhibition of NOX2 signaling through the attenuation of histone deacetylase activity in T cells following sepsis-induced kidney injury [23]. In an animal model of acute kidney injury (mice that underwent bilateral renal ischemia-perfusion), Andrade-Oliveira *et al.* [24] assessed the impact of acetate, propionate and butyrate on renal function following ischemia and supplementation with any one of the SCFAs ameliorated the degree of renal injury. Outcomes included reduced serum creatinine levels, blunted pro-inflammatory cytokine/chemokine production and decreased necrotic scores in kidney tubular epithelial cells. These effects were most prominent following acetate supplementation and were also observed when acetate-producing bacteria were given by daily gavage in place of chemical supplementation.

Furthermore, it has been shown that the feeding of a diet high in fermentable fibrous material (e.g., amylose maize resistant starch or HAMRS2) mimics the beneficial effects of ameliorating kidney disease in a similar way as observed with SCFA supplementation (reduced declines in renal function, less interstitial fibrosis and tubular damage, and reduced activation of inflammatory and fibrotic pathways) [25]. Alongside these clinical improvements, high-fiber diets induce changes in circulating gut metabolites through the upregulation of SCFA production and decreases in *p*-cresol, indoxyl sulfate and TMAO [26,27]. This demonstrates a multimetabolite interaction between the metabolic processes of the gut bacteria and host health, with potential application of promising nutritional strategies to elevate bacterial SCFAs, reduce build-up of uremic toxins and subsequently improve disease characteristics.

Despite the potential benefits documented with SCFA supplementation, findings are predominantly restricted to *in vitro* and *in vivo* models of renal disease. Importantly, there is a current lack of clinical research investigating the potential of circulating SCFA measurements to serve as biomarkers in diagnosis, prognosis and therapeutic monitoring of renal patients. Further study would therefore seem warranted. The routine clinical measurement of systemically absorbed gut SCFAs offers the potential to develop clinical risk stratification tools that may then lead to targeted treatments to slow CKD progression. This could be in the format of dietary (e.g., high-fiber), medicinal (e.g., SCFA supplementation) or biological (e.g., probiotics) interventions. Further research in this area will provide insight on whether increasing circulating SCFAs provide any direct clinical benefit, which ultimately could result in a new therapeutic approach for people with CKD.
